# The Learning Cycle method: an approach to rapidly bring evidence to the point of decision-making for health system planning

**DOI:** 10.1186/s12961-026-01453-w

**Published:** 2026-02-14

**Authors:** Morgan Price, Amanda Frazer, Elka Humphrys, Angela Randall, Tlell Elviss, Sarah Fletcher

**Affiliations:** 1 Faculty of Medicine, Department of Family Practice, University of British Columbia, Vancouver, Canada; 2 Faculty of Medicine, Island Medical Program, University of British Columbia, Victoria, Canada

**Keywords:** Learning health system, Action research, Rapid cycle, Knowledge mobilization, Implementation, Learning Cycle, Quality improvement, Primary care

## Abstract

**Background:**

Health systems often struggle to integrate local and published evidence into timely policy decisions, particularly in complex and rapidly evolving environments such as primary care. While learning healthcare systems (LHS) principles advocate for continuous learning through real-world application, practical methods for operationalizing this without infrastructure can be challenging. To address this gap, we developed the Learning Cycle method in collaboration with the British Columbia Ministry of Health to help drive action-oriented, evidence-informed change and support decision-making.

**Methods:**

A Learning Cycle is a low-barrier, rapid (8–12 weeks) knowledge mobilization approach that collects evidence and brings it into policy and/or planning decisions through participatory action methods. The process is initiated by a business owner who has a question that guides the focus of the cycle. The Learning Cycle Lead then manages the cycle, which has two phases: (1) a learning phase – where mixed sources of evidence are rapidly synthesized to address partner defined questions and (2) an action phase – where results are shared in a structured, constructive workshop with decision-makers to facilitate evidence-based planning. The business owner provides input and takes the feasible change ideas into implementation.

**Results:**

To date, 25 Learning Cycles have been completed by the British Columbia (BC) primary care Innovation Support Unit (ISU). Four illustrative case studies are presented to demonstrate the method’s flexibility, responsiveness and utility in policy development, planning and system redesign. Contributors to successful Learning Cycles include securing buy-in and continued engagement from the business owner, aligning the cycle to a clear initial question, timing it right to a decision point and adapting to system conditions.

**Conclusions:**

The Learning Cycle model demonstrates adaptability to different contexts, capacity to rapidly align evidence with decision-making needs and potential to embed learning functions across jurisdictional levels. The Learning Cycle is a lightweight method to mobilize evidence into policy and practice with minimal infrastructure, thus making it suitable for low-resource or structurally diffuse contexts, and complements more robust LHS approaches. Its structured yet flexible design advances core LHS strategies – continuous improvement, evidence integration and stakeholder co-production – making it a promising model for responsive and equity-focused health system transformation.

## Background

### Learning healthcare systems

Learning healthcare systems (LHS) were proposed to support the change needed to address gaps in quality of care [1, 2]. LHS develop a whole-system approach to embedding evidence into continuous quality improvement in a patient-centred and sustainable manner [3]. The foundations for an LHS include: robust infrastructure for consistent health data capture, healthcare improvement targets and a supportive policy environment [4]. LHS are characterized by continual improvement and innovation [5, 6], where evidence generation and application are embedded into practice [3, 6, 7]. LHS have been implemented in a number of settings [7] and support evidence synthesis, stakeholder dialogue and implementation monitoring as employed by the Swiss Learning Health System [8]. However, their robustness can also make LHS difficult to implement. The LHS Action Framework highlights key features of an LHS but also highlights challenges or so-called brakes that slow down an LHS, including: disconnected systems, cross-organizational barriers, limitations in the speed of evidence generation and gaps between evidence generation and implementation [3]. Further, LHS implementations are often exacerbated by decision-making processes and capacities that are not well-aligned with the timelines of evidence creation [7, 9].

Rapid-cycle research methods are essential in complex dynamic systems, where responsiveness and an understanding of local and system context are critical for sustained impact [7, 10]. In an LHS, this typically requires the core infrastructure highlighted in the Learning Action Framework [3]. There are open-source tools such as i2b2 that reduce implementation efforts [11] of core informatics infrastructure, but full implementation requires substantial and sustained effort [6, 7].

Beyond data and technical infrastructure, other so-called fuel and accelerants[3] for LHS include leadership, expertise, engagement with partnerships (with, for example, patients and families, communities, Indigenous peoples, equity deserving groups, and health workforces), scientific expertise, implementation and decision support, learning networks, and funding. But these components of LHS can take time and considerable effort to ignite. What happens when these are not (yet) a core of a health system? How can a health system start learning? There are lightweight processes such as local quality improvement activities and learning networks [12]. These focus on answering clinical questions and generally do not directly support answering policy questions. Policy questions are often answered with a rapid review or literature scan and little available direct evidence [13]. To start to address this policy gap, in the context of public health, Kilbourne et al. (2024) introduce what they call Translation to Policy (T2P) Learning Cycles in public health, which incorporate LHS infrastructure and work to align policy with national public health priorities (based in the United States Veterans Health Administration)[13]. In primary care and at the local community level, there remains a dearth of approaches to support the integration of timely evidence into policy-making.

### Primary care

While primary care is the bedrock of a high-functioning health system [14], in many countries, such as Canada, the structure of primary care makes it challenging to implement an LHS. Many primary care systems have three of the identified brakes in the LHS Action Framework [3] that make it hard to implement a comprehensive LHS: capacity gaps, complex privacy legislation and unclear or diffuse governance. Internationally, primary care is at or beyond capacity, and Canada is no different [15, 16]. The combination of private providers and public healthcare in Canada means multiple organizations and sometimes multiple privacy legislations negatively impact creating a robust data infrastructure. Data are often siloed within practice-level electronic medical records, and data quality is a long-standing issue [17]. Countries are adopting team-based care (TBC), widely recognized for the potential to improve primary care access and sustainability for patients and providers [18, 19]; however, TBC can be difficult to implement [20] and would benefit from an LHS to guide implementation. In BC, there has not been an LHS for primary care, and there is a growing need for timely, context-sensitive learning methods that inform policy and planning decisions.

#### Fostering nascent LHS – Learning Cycles

In this paper, we introduce the Learning Cycle method, a lightweight process developed to rapidly include local and external evidence into policy planning decisions, before an LHS is operational. We provide four examples as case studies from British Columbia, drawn from our experience in primary care. The examples showcase the diversity of Learning Cycles in terms of scope, audience and output, and we highlight how Learning Cycles can fit into larger change initiatives. But before describing the method, how do Learning Cycles compare with larger-scale LHS approaches?

## Methods – ISU Learning Cycle approach

The Innovation Support Unit (ISU) Learning Cycle method is a rapid, participatory knowledge mobilization approach grounded in principles of action research, developed by the University of British Columbia’s Innovation Support Unit (ISU) in partnership with the BC Ministry of Health. The aim of this collaboration was to support primary care policy and planning decision-making in a time of significant change in the BC primary care system. The Learning Cycle aims to embed local evidence into policy and planning decisions to support informed decision-making within complex, sometimes rapidly evolving policy environments.

A Learning Cycle has two explicit goals:

1. To rapidly discover and synthesize evidence (published and local), prior to a planning or policy decision point.
2. To facilitate the rapid integration of the synthesized evidence into decision-making.

### Overview of the Learning Cycle method

**Fig. 1:**
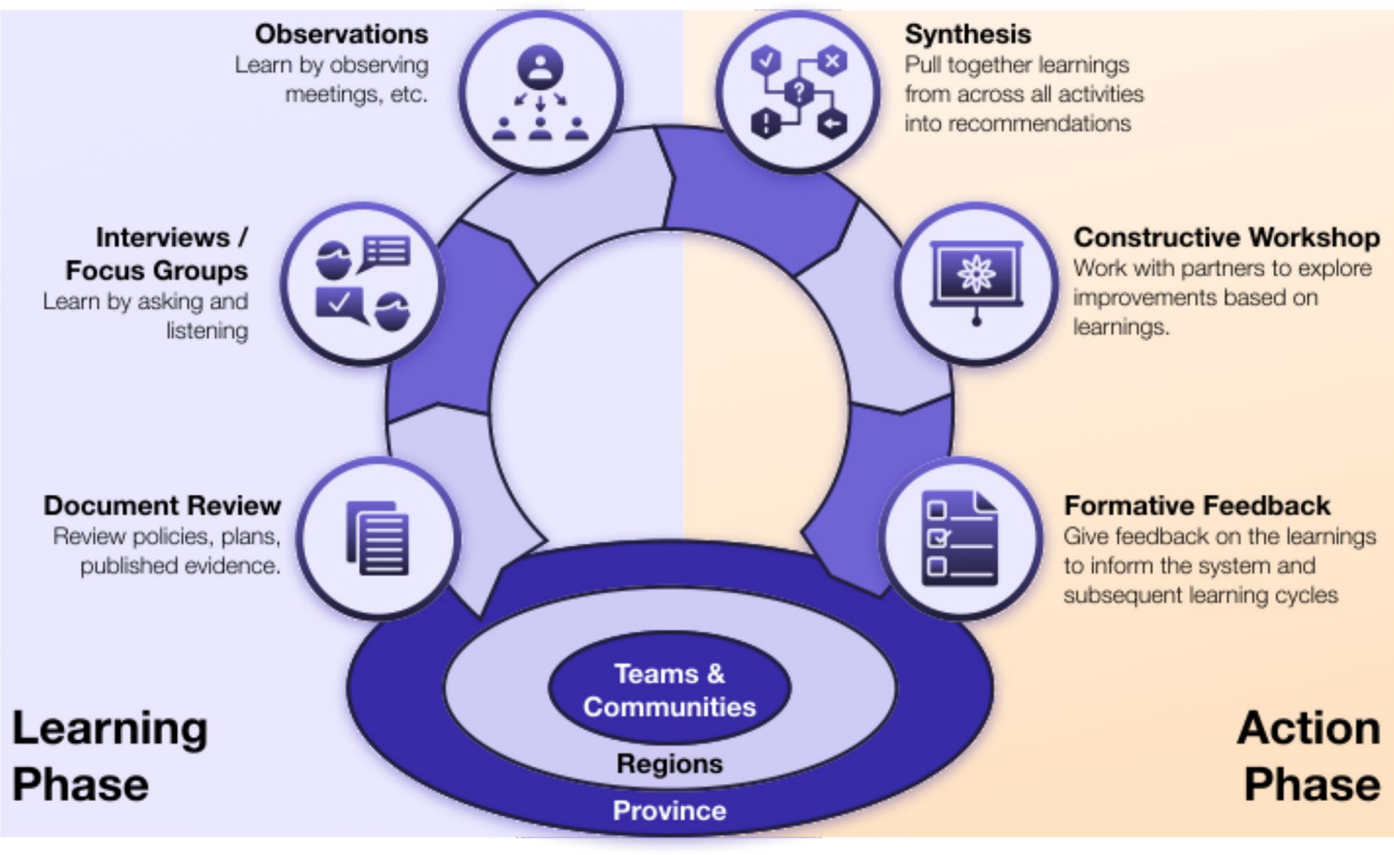
The archetypal Learning Cycle with a clear learning phase and action, or construction, phase

A Learning Cycle is driven by a business owner, often a policy-maker or health system planner. Typically, they have a time-sensitive decision to make on policy or implementation of policy that could benefit from evidence-informed input. The Learning Cycle Lead then helps craft an answerable question and designs a pragmatic, time-boxed Learning Cycle – typically 8–12 weeks – to generate sufficient evidence to assist the business owner with their question, timed to bring that evidence to the point of decision-making. Depending on the scope, the Learning Cycle Lead recruits team members to help with the Learning Cycle. The Learning Cycle proceeds in two main phases (Fig. 1):

- Learning phase: evidence gathering (local and/or published).
- Action phase: findings are synthesized and used to co-design actionable solutions.

The Learning Cycle is low-barrier and flexible, designed for use at multiple system levels (e.g. provincial, regional, local) and adaptable to a wide range of topics – from service design to policy development. It operationalizes core LHS functions by rapidly converting performance- and context-derived data into actionable insights (data-to-knowledge) [13], integrating those insights into decision-making (knowledge-to-practice) and providing feedback that informs future cycles.

### Learning phase

The learning phase includes data collection from two to three information gathering methods. These data sources are often readily available, requiring little infrastructure:

1. Document review: review of relevant, existing policies, reports, proposals and evaluations. This can also include literature scans or environmental scans: rapid reviews of academic and grey literature that align with the question. Identify evidence, best practices, related policies, new models or innovations that may be applicable to the Learning Cycle question.
2. Interviews/focus groups: semi-structured interviews or small focus groups from a diversity of roles in the system that are relevant to the scope of the question (e.g. regional planners, clinicians, policy-makers, community partners, patients, Indigenous voices). These are typically identified with the business owner. Ensuring there is a diversity of perspectives, paying close attention to missing voices and perspectives and applying a lens that is informed by the principles of justice, equity, diversity and inclusion to the process. Interviews explore impact of current policies or plans, challenges, barriers and areas for feasible improvement.
3. Meeting observations: if there are any meetings that can be observed, we encourage this as part of a Learning Cycle. Observation provides a different understanding of process or context related to the topic. These could be internal planning meetings or presentations with stakeholders, among others.

Depending on the question, resources and infrastructure, additional methods and data sources can be helpful. Surveys or using existing health data can be helpful if feasible to complete.

Initial synthesis (see below) typically occurs iteratively through the learning phase to allow for snowball-type approach to information gathering.

### Action phase

The action phase centres around a constructive workshop and supports the business owner to make evidence-informed decisions and initiate change in a collaborative, co-design process that is grounded on the data gathered.

#### Synthesis

Findings from the learning phase are synthesized into four to eight key themes. Synthesis is carried out by the Learning Cycle team and reviewed with the business owner. Key themes are pulled into a short report and/or presentation that can be easily shared. The synthesized learnings are then drawn into the design of the constructive workshop.

#### Constructive workshop topic

In collaboration with the business owner, one or two themes are selected for action. Selection is based on feasibility, potential impact and decision-making need. The selection of the topic for a constructive workshop is guided by the XYZ Formula:

Theme *X* is interesting to participants/the system because of *Y*

It could result in a feasible change such as *Z*.

This formula helps ensure that the workshop topic is relevant (*X* and *Y*) and action oriented (*Z*). The topic is generally related to the original question but may well be an emergent finding that is more focused and relevant to the decision point of the business owner than the original question.

#### Constructive workshop participants

Participants who are decision-makers related to potential changes to be discussed in the workshop are recruited. One of the keys to the success of a constructive workshop is the participation of partners who understand the issue first-hand, have the authority to act on decisions emerging from the workshop and are involved in the upcoming point of decision-making. As with the interviews, we encourage diversity here as well (workshop participants may or may not be some of the people interviewed in the learning phase).

#### Constructive workshop

The 2–3-h facilitated workshop leverages design thinking techniques to generate change ideas, select preferred options, prototype a specific feasible improvement and agree on a course of action to implement that change. The specific design thinking techniques will vary for each workshop and align with the goal. If you are wanting to co-design a change in process, consider persona-based journey mapping or business process modelling. For choosing between options, tools for collaborative prioritization and decision-making are used, such as dot voting and ranking [29]. Discussions and decisions are documented in real time by facilitators so that all participants can see that their ideas are captured. At the end of the workshop, decisions and actions are confirmed with participants, ensuring that outputs are feasible, clear and actionable.

#### Formative feedback, outputs and knowledge mobilization

Within days, the Learning Cycle Lead shares a brief report summarizing workshop decisions, key themes and visual artifacts from the session. This rapid turnaround of formative feedback from the session maintains momentum and supports follow-up implementation of the ideas.

This feedback can include recommendations for decisions (e.g. to be taken to governing group) and recommendations for actions (e.g. starting a project/program to address a learning). Recommendations should be made about next actions, and the Learning Cycle Lead, especially if they are embedded in the system, can work to ensure implementation of ideas.

Depending on the role of the Learning Cycle Lead, they may move the learnings to action and measure impacts of the change. In the case of the authors, we are an external academic group, and the responsibilities for system change are with the business owner.

Another output can be additional Learning Cycles. Follow-on cycles can be more homed in topics within the same area of the first Learning Cycle or they could be an emergent topic informed by the findings of the initial Learning Cycle.

Further knowledge mobilization activities are conducted with the business owner and may include sharing findings with related groups or communities, regional leadership and provincial/jurisdictional governing bodies. These engagements share the insights and changes from the workshop and ideally may also spark follow-up questions and future Learning Cycles, building the system’s interest in the value in learning.

### Learning cycle variations and flexibility

The methods above outlined the archetypal Learning Cycle; however, practitioners are invited to adapt the method to their context. Example variations include:

- Focus groups instead of interviews to gather local evidence.
- Greater focus on external interviews and environmental scans when local knowledge is limited.
- The integration of different methods to rapidly collect information and feedback (e.g. surveys, photovoice).
- Multiple workshops to explore findings and construct change plans.

Adaptations are made to suit the context, stakeholder needs and timeline – while maintaining the core principle of bringing timely evidence to the identified point of decision-making.

Follow-up evaluations, meetings with the business owner and interviews with stakeholders can help understand the impacts and barriers to impact from the Learning Cycle. The evaluation should be tailored to both the scope/size of the Learning Cycle and related to the nature of the changes.

The University of British Columbia (UBC) Ethics Committee reviewed the overall learning cycle approach and deemed it to be quality improvement as it supports internal system learning.

#### Comparing Learning Cycles to comprehensive LHS models

While the Learning Cycle model described below has similar goals to the larger LHS models, there are distinctions that highlight the unique applicability of the method.

**Grounded in community-identified questions to support a deliberate fusion of policy practice and research (versus clinical question, research-driven systems)**

Many LHS models [21, 22] begin with an exploration of a clinical delivery system and then seek to identify areas for improvement and bridge out to policy. In contrast, the ISU Learning Cycles were designed to serve policy partners’ or community partners’ immediate planning questions. By working at the policy or planning point of decision-making, ISU Learning Cycles improve likelihood that the answers align with current issues and supports faster translation into action for system-level decisions.

**Driven by short, action-oriented focused cycles (versus comprehensive, data-heavy iterations over extended periods of time)**

While the idea of rapid-cycle research is not new [3, 5, 9], and is a goal for LHS, many LHS depend on a core infrastructure and policy framework with longer time to set up and establish. For example, much of the work that draws on electronic health record (EHR) data [6, 7, 23] requires standardization, data quality assessment and clean-up. There are governance decisions and typically various new quality and oversight committees that are established as part of the development of LHS. These are valuable and needed for whole system change. Learning Cycles complement this approach, particularly before governance and data structures are in place; relying on agile and mixed methods, Learning Cycles can rapidly triangulate evidence and bring it to the point of decision-making where there may be little evidence available prior to establishing a more robust LHS.

**Embedded engagement strategies through a focus on facilitated co-creation of next actions (versus a technological infrastructure focus)**

Developing LHS often focus first on building the system-wide infrastructure, including data platforms, information pipelines, analytics and governance processes [13]. In contrast, the ISU Learning Cycle intentionally requires less infrastructure to start. They take a more human-centred design approach with facilitated stakeholder engagement that support data collection, interpretation of findings and shaping of solutions. While Learning Cycles could be embedded into mature LHS as they provide relational, facilitation-based, persona-driven methods to navigate power dynamics, hidden assumptions and context, they were originally designed and applied in a system lacking established infrastructure as a way to initiate the culture shift towards LHS, with only small investments in human capacity and time.

**Design thinking integration (versus research-to-practice models)**

ISU Learning Cycles explicitly draw on design thinking approaches to tackle so-called messy problems. Tools such as circle of care mapping and patient or provider personas are used to help decision-makers see the system in new ways to uncover hidden assumptions and power dynamics [24–26]. Traditional LHS have established data collection mechanisms and feedback cycles, whereas Learning Cycles are perhaps a more nimble or flexible open process with multiple entry points. Learning Cycles are intentionally nonlinear and are suited for exploring emergent problems or questions that might fall outside the scope of an LHS. As such, they are designed to assist with complex or wicked problems.

**Built with focus on equity, diversity, inclusion and justice principles (versus optional ethical considerations)**

In early LHS frameworks, equity was often a parallel consideration. More recently, the need to intentionally focus on equity perspectives in the context of LHS has been increasingly recognized. Menear et al. (2019) include it as a pillar, and Reid et al. (2024) present an applied framework for a comprehensive LHS approach to advance both population health and health equity. They draw on excellent work in this space that has been carried out to support the inclusion of equity perspectives in LHS [9, 27, 28]. Similarly, Learning Cycles also integrate justice, equity, diversity and inclusion, from document reviews to interviews (paying close attention to voices that are typically missing from evidence-gathering) to facilitation and analysis.

The whole system approach of robust LHS frameworks, such as the LHS Action Framework, are anchored in data and policy infrastructure and embedded evaluation, which works well in environments with high IT and analytics capacity. However, it can be challenging to implement this approach quickly or in resource-constrained settings. While being grounded in action research principles, the Learning Cycle process takes the opposite approach and represents a pragmatic, single-question approach to generating learnings to support policy improvement and health system decisions. The Learning Cycle method could serve as a replicable micro-LHS model for other settings seeking practical approaches to operationalize learning and improvement to inform policy questions.

## Results

Overall, 25 Learning Cycles were completed in BC between 2018 and 2025. These cycles addressed a range of policy and planning questions related to team-based care, population health services, capacity planning and workforce development. Evaluation has primarily focused on participant feedback on application of materials from the constructive workshop in planning or follow-on activities. Early feedback from partners and participants suggests that the Learning Cycle contributes to timely evidence synthesis, strengthens engagement and supports implementation of locally relevant strategies. Several outputs have been embedded in planning documents, service redesign initiatives and evaluation frameworks.

Four illustrative cases are described in detail in the following section. These examples were selected to demonstrate the Learning Cycle’s adaptability across contexts and its ability to support actionable learning at multiple levels of the system. Some of these examples showcase the potential for rapid integration of evidence into policy or health systems planning, others highlight lessons learned.

### Service plan planning – it is not (always) rocket science

#### Reviewing the BC primary care network service plan development process

**Context:** The first Learning Cycle highlights rapid, timely learning informing program changes. BC had embarked on its new primary care strategy that involved creating over 90 primary care networks (PCNs) across BC [30]. At the time of this Learning Cycle (November 2018) BC’s primary care program had just concluded their first round of PCN service plan applications (a process of over 120 days). The whole process of development and approvals was more work and took months longer than expected. Changes were needed to the service planning process before opening a second wave of funding, which was about 9 weeks away.

**Learning Cycle questions:** What can the BC Ministry of Health learn from the wave 1 PCN service planning process to improve wave 2 before launch?

We completed focus groups and interviews with community representatives who applied for PCNs, policy-makers and decision-makers involved in supporting the PCN planning and evaluation (18 participants), and reviewed key documents (primary care policy, planning process documents, example plans). Key learnings highlighted a desire to be more agile, the need for more guardrails and parameters in the process, and challenges with the timeline as well as communication and engagement gaps with and within communities. These were underscored by a desire for more data-driven planning.

**Constructive workshop:** The workshop was with primary care ministry leadership. The workshop focused on two learnings where we thought there could be feasible change: the need for guardrails and the desire to have an agile process. We opted for a timeline activity and facilitated the co-creation of a new service planning process. Participants identified high-risk decisions and ensured that those were addressed earlier in the process instead of when the final plans were submitted. The new timeline front-loaded higher-risk decisions with built-in checkpoints for early identification of challenges with communities. By co-designing and prototyping a new timeline, leadership had worked through rationale and feasibility in the workshop.

**Output:** The revised service plan process from the workshop was implemented by the government during wave 2 of PCN service plan launch, 5 days after the workshop.

**Take away:** Timing matters and simple changes can have considerable impact.

### Everyone is pivoting – a learning cycle at the start of the pandemic

**Context:** In March 2020, at the start of the coronavirus disease (COVID) pandemic, the UBC Department of Family Practice (herein, the Department) was unsure of how to support their faculty and staff. The Department has a small core staff and academic faculty and several thousand clinical (e.g. teaching) faculty working in primary care across BC. Everyone was making massive changes on the basis of the declaration of the COVID pandemic, including moving to virtual care, working from home, among others. The ISU had capacity as many of its projects were on hold. The Learning Cycle method was well-suited for the rapid change that happened at the start of the pandemic.

**Learning Cycle question:** What supports from the Department would be the most useful for our faculty and staff?

This Learning Cycle began with a survey of clinical faculty in primary care to broadly collect concerns and needs. Synthesis included summarizing the needs then identifying where work was already underway by others and highlighting gaps that could benefit from support. Some of the gaps identified included: (1) supporting primary care providers in transitioning to virtual care and supporting teams (clinical and academic) to virtual teamwork; (2) gaps in knowledge and the need for a one-stop shop for information; and (3) lack of up-to-date information channels beyond public health that were tailored to primary care.

**Constructive workshop:** Where can the ISU rapidly pivot to best support the Department and the primary care system? The constructive workshop mapped the needs with the skills in the ISU. The goal was to have a tactical list of supports the ISU could develop.

**Outputs:** A list of support projects that could be linked to identified needs from primary care clinicians, faculty and staff was created. This directly led to the development of several projects, including: webinars on “How to work better virtually” for staff and faculty and 1:1 consultations; creation of a “Primary Care RNs Virtual Care Checklist” to support nurses in knowing what is safe to do remotely (and how); Primary Care in a Pandemic podcast to provide tactical advice to our primary care providers (this evolved into the TeamUp podcast); and the development of the national “Covid Toolkit” website to support practices planning safe, mass immunization clinics when immunizations were available. This Learning Cycle also led to the development of the immPACC project (https://isu.ubc.ca/about-us/past-projects/immpacc/) – a multi-year project focused on supporting immunization planning in Western Canada [31].

**Take away:** Learning Cycles can be used as an internal tool, and a small focused rapid cycle can identify a broader set of new projects or outputs.

### Learning cycles to support primary care evaluation planning

Context: This case study comprises two connected Learning Cycles and highlights how learning cycles can build on each other. The business owner was BC Ministry primary care leadership. They identified the need to develop a clear plan to measure the impact of the changes in primary care in BC as the new PCN strategy was being implemented. There were program implementation metrics but not a clear evaluation framework or plan.

**First Learning Cycle questions:** What could be the scope for a feasible and meaningful evaluation plan for primary care networks? Who should be involved in co-developing it?

To explore this question, we sought local information (interviewed 33 stakeholders, reviewed existing policy and planning documents, observed meetings). We scanned published and grey literature on primary care evaluation frameworks and created a list of 28 common evaluation domains (e.g. access to care, patient experience, provider experience) on the basis of 13 relevant frameworks.

**Constructive workshop:** Primary care leadership from government, health regions and the BC Family Practice Services Committee participated in the workshop. The workshop focused on (1) prioritizing the evaluation domains and (2) creating a stakeholder matrix for evaluation planning. A third output of the learning cycle, a proposed co-development process, was presented for feedback.

The three outputs from the Learning Cycle informed the next stages of the provincial primary care evaluation planning process. It also led to a second Learning Cycle.

**Follow-up Learning Cycle:** One of the challenges with focused, time-boxed projects such as Learning Cycles is that competing priorities can take away from the project’s momentum. The first Learning Cycle was completed just before the pandemic. After the initial wave of COVID, the government asked for a second evaluation Learning Cycle to help restart their planning process. This Learning Cycle used the planning process from the initial cycle and started to review evaluation tools to measure provider experience, one of the priority areas identified in the first cycle. The second evaluation Learning Cycle’s constructive workshop focused on stakeholder-led prioritization of indicators and co-design of a toolkit for the evaluation of provider experience in the context of team-based primary care.

**Outputs:** A shortlist of 16 high-potential tools, with a finalized list of evaluation questions and indicators informed by key knowledge holders as well as a tailored the approach to evaluation for the BC context.

**Key takeaway:** Tightly scoped cycles can scaffold larger initiatives, and a focused cycle can catalyse broader system design – especially when paired with intentional co-design-focused engagement opportunities for key stakeholders.

### Attachment or access? Making hard decisions in community

This Learning Cycle took place in the winter of 2022–2023, and the business owner was a local division of family practice in BC. Divisions of family practice support a geographic area of primary care providers. In rural settings, a division can span several towns. In this case, the division wanted to explore how to adapt primary care services, given the persistent capacity gaps and significant numbers of unattached patients in their area.

**Learning Cycle question:** Should the division do something different to provide more services to unattached patients?

In the learning phase, the team read division plans and reports as well as published papers that were informing the community’s planning process. After interviews with the division leadership, it became clear that they needed more feedback from their members on the tensions related to attaching more patients versus providing good quality care to the patients currently attached versus providing more episodic care for unattached patients. They wanted to have a broader, facilitated dialogue about the current challenges, trade-offs and the impact on their providers.

**Constructive workshops:** The Learning Cycle Lead opted to shift from a single workshop to a series of constructive workshops, gathering data from each community in the region while facilitating discussion on the implications of the options. A set of nine even-over statements were created to guide the conversation. Even-over statements [32] are a design tool that creates a series of forced-choice comparisons that have difficult trade-offs, such as “provide good enough care to all versus provide high-quality care to those attached”. Participants must individually pick one option (forced choice) anonymously and the results are aggregated. A set of even-overs helps explore relative priorities and can move groups forward that avoid making tough decisions. For each workshop, the participants completed the even-over individually and then discussed results and the pros and cons of each option.

**Outputs:** In this Learning Cycle, the dialogue and engagement in constructive workshops were seen as an important output. The results of the even-overs for each community and the summarized discussions informed local planning for the division.

**Key takeaways:** A Learning Cycle can be used to gather information from a broad cross-section of stakeholders and can accelerate co-design to inform community planning.

## Discussion

This paper introduces the ISU Learning Cycle as a practical method for embedding learning into real-time planning and policy processes. Grounded in the principles of learning health systems (LHS) [7] and participatory action research [33], the approach facilitates context-sensitive knowledge mobilization within a compressed timeline. In this section, we reflect on the method’s value, and on the differences between larger LHS models and local policy-level focus of the ISU Learning Cycle method.

Many LHS approaches are comprehensive frameworks that provide a powerful system-level architecture for embedding learning in healthcare [3, 9, 22], which we support. The LHS Action Framework provides an excellent whole system approach to LHS development, providing the system-level architecture for embedding learning into healthcare. To support the practical integration of research into health systems, a number of LHS models have outlined research-to-action cycles [3, 9, 13, 23]. There are pragmatic approaches that focus on quality-improvement-type activities that can be applied with little infrastructure. However, there are fewer tools for nascent LHS where the focus is on supporting policy decision-making [13] and in systems that lack some of the so-called fuel and accelerants such as established learning networks, partnerships, leadership or funding that would hinder establishing a robust LHS. ISU Learning Cycles were developed to fill this niche, complementing and potentially catalysing the development of more robust LHS systems, and applied in British Columbia’s evolving TBC primary care system.

LHS theory rests upon a cyclical learning process, and there are many variations of the stages of these models in literature [6, 7, 9, 13, 22]. All models are broadly in line with a problem identification–design–implement–evaluate–adjust (and disseminate) learning process [9] or a data to knowledge–knowledge to performance–performance to data cycle [13]. The ISU Learning Cycle compresses this, emphasizing agility, ease of uptake and co-design to support targeted evidence generation and action-oriented policy improvement support. Where scope of most LHS research and interventions are often larger, the ISU Learning Cycle method has been intentionally designed to enable local, responsive planning and equity-oriented decision-making with minimal infrastructure, which was suitable to the primary care context.

Policy is not decided solely on scientific evidence but also considers other factors such as the overall policy/political agenda, capacity, other needs and appeal to constituents [34]; thus, an LHS that seeks to improve policy needs to take a pragmatic approach to support this complex decision-making framework that can be tacit. Researchers who seek to engage in policy need to consider these factors, engage humbly and flexibly, and build relationships [35]. One can engage policy-makers through co-creation of evidence and decisions [36]. Learning Cycles, on a small scale, seek to do this through the constructive workshop. Selecting different design-thinking methods for the workshop, it can serve to highlight and make explicit the tensions and trade-offs between competing factors for the specific policy focus of the Learning Cycle. For example, the use of even-over statements comparing different tensions is one approach to include in the workshop to develop consensus on competing priorities on a topic.

### Lessons learned from implementing learning cycles

These learnings were collected from reflections by the authors who have participated in each learning cycle.

#### Starting small, building trust

Our clear takeaway with Learning Cycles is that starting small can be a strength. Short, well-scoped Learning Cycles with a clear output are easier for busy decision-makers to agree to. The smaller scale and clear timeboxing of Learning Cycles creates a so-called safe-to-try environment that can be especially useful when relationships are developing or when there might be high degrees of risk aversion to larger system-level innovations. Rapid, successful projects help establish trust, support experimentation and allow for adaptation to emerging questions.

#### Small can be forgettable

Learning Cycles’ strength, their small size, is also a key limitation. With their narrow focus and time-bound nature, there is a risk that the outputs may not be integrated into the planning process or that they are seen as a one-off answer and not the start of a learning system. Even well-received cycles may lose visibility over time without a clear knowledge mobilization and follow-through plan with partners. The barriers that hinder establishing more robust LHS (e.g. lack of funding, learning networks, capacity for change) also impact Learning Cycles, and we have found that there may be capacity to complete a Learning Cycle and incorporate the findings into a policy or practice change but not the capacity to use the Learning Cycle as the start of a fly wheel to begin building a more robust LHS. One way to mitigate this is to propose a series of directly related Learning Cycles so that momentum builds and components of infrastructure are actively constructed through the cycles with decision-makers over time (e.g. engagement with partners, become a standing LHS committee or learning network).

#### Small does not fit all issues

Many problems are too large for Learning Cycles. Questions that are too broad, lack a clear decision point or require more effort than what can be applied in 8–12 weeks are not good fits for this method. To use Learning Cycles for larger questions, they need to address specific subquestions that are embedded in larger projects.

A limitation of small projects is that the funding is targeted, and so the evaluation of the longer-term impact of the Learning Cycle is often not in scope. Further, it can be challenging to extract out the specific contributions of each piece of what becomes a complex policy intervention.

#### Decision-maker buy-in is crucial

Successful Learning Cycles require buy-in from the business owner. First, the business owner needs to be invested in the upcoming decision (policy, program change, among others) that the Learning Cycle is informing. Next, the business owner should have the ability to translate the results into actions. Lastly, keeping engaged with the business owner helps the Learning Cycle stay timely. If a Learning Cycle is late, even well-crafted recommendations will not have traction if they arrive after key decisions have been made.

#### The right people in the room

In the learning phase and more so for the constructive workshop, what really matters is getting the right people in the room. For change to occur, workshop participants must have both the practical experience to contextualize issues and the authority to act on proposed solutions. When this balance is off, momentum stalls post-Learning Cycle.

#### Timing and coordination

With a Learning Cycle that is tightly connected to a decision, small delays can result in missing the opportunity to inform that decision. Changes in timing on a decision can also mean a Learning Cycle misses the decision point. Careful project management and flexibility to adapt to ensure learnings are shared at the right time is key.

#### A little structure can go a long way

Learning Cycles can offer a structured framework to tackle reactive chaos. In times of rapid change and uncertainty (such as in the pandemic), it can be easy for policy-makers and healthcare leaders to feel overwhelmed and make rapid decisions without reflection. The Learning Cycle method provides a process for guiding strategic pivots when a larger process could not be implemented. Urgency and uncertainty can create opportunities for rapid learning and responsiveness.

#### Build momentum beyond the cycle

At the same time, one cycle does not make a learning system. While a single Learning Cycle can produce timely insights and a tangible output, broader change requires a strategy for sustaining momentum. This can include sharing learnings more broadly, creating the necessary processes to measure impact of the changes, adding follow-on Learning Cycles and ideally using the early momentum to catalyse the bigger discussions towards a more fulsome LHS. Without follow-up or links to broader structures, Learning Cycles alone cannot enable larger change, and the reason (lack of infrastructure) that makes a Learning Cycle appropriate as a place to start is also a risk for why the changes may not be implemented or sustained. We recommend embedding into Learning Cycles, as part of recommendations and knowledge-mobilization components of the LHS, some fuel and accelerants that could be adopted in the system. For example, a committee or group that is focused on the recommendations and follow through of the Learning Cycle could be tasked with starting a learning network, or the engagement created through the interviews and workshop with partners could be extended into a standing committee.

#### Results can have future value

Even though Learning Cycles are targeted and focused on a specific question, we have been surprised that the reports have been reviewed months and sometimes years later to find some evidence or a theme from the report that has become relevant again when context shifts or a new change is being considered. Themes that were identified in a Learning Cycle but not actioned in the workshop can inform future decision points.

#### Equity requires time

Embedding equity and inclusion meaningfully into each cycle takes effort and intention. It requires more time to build meaningful relationships than a single Learning Cycle. Developing meaningful relationships requires relationship development well before a Learning Cycle, and not only as a time-limited engagement during a short project. In addition, within a Learning Cycle, equity cannot be an afterthought, especially with short timelines. The Learning Cycle Lead needs to be deliberate about who is interviewed, which and how sources are collected, which issues are surfaced through synthesis, who is invited to the workshop and how findings are shared.

Future research could include a more formal evaluation of the effectiveness of the ISU Learning Cycles, as well as an exploration of the applicability of the method in more diverse contexts and, particularly, in otherwise under-resourced settings.

## Conclusions

Learning Cycles offer a practical, lightweight method for bringing evidence into decision-making where the infrastructure for an LHS is only emerging, such as the authors’ experience with the diffuse nature of primary care systems in Canada. Learning Cycles facilitate a focused, miniaturized LHS on a specific question to help bootstrap evidence-based decision-making for policy-makers and planners. They help policy-makers and system leaders by generating insights and actions, bridging the gap between aspiration and implementation in healthcare improvement.

The case studies demonstrate that Learning Cycles can catalyse broader initiatives. At the same time, challenges remain, such as sustaining momentum, right-sizing questions, coordinating participation within short timelines and ensuring integration into wider system learning efforts. These can be addressed as the healthcare system matures their LHS approach.

## Data Availability

No datasets were generated or analysed during the current study.
